# Primary Endometrioid Adenocarcinoma in the Right Fallopian Tube Mimicking Female Adnexal Tumor of Probable Wolffian Origin: A Diagnostic Pitfall

**DOI:** 10.7759/cureus.90168

**Published:** 2025-08-15

**Authors:** Fei Han, Sayeda Humaira, Michael Dardik

**Affiliations:** 1 Pathology, Cooperman Barnabas Medical Center, Livingston, USA; 2 Pathology and Laboratory Medicine, Cooperman Barnabas Medical Center, Livingston, USA

**Keywords:** diagnostic challenge, endometrioid adenocarcinoma, fatpwo, immunophenotype mimicry, pax8 negative

## Abstract

Female adnexal tumor of probable Wolffian origin (FATPWO) is a rare neoplasm that morphologically mimics endometrioid carcinoma and lacks specific immunomarkers or molecular alterations, posing a significant diagnostic challenge. We report a case of a 51-year-old woman with an adnexal mass initially diagnosed as endometrioid adenocarcinoma arising in the right fallopian tube. The tumor closely resembled FATPWO both morphologically and immunophenotypically, exhibiting loss of PAX8 and cytokeratin 7 expression, with no identifiable recurrent molecular alterations. However, the extensive tubular and focal papillary growth patterns observed are uncommon in FATPWO. Notably, PAX8 negativity can occur in endometrioid carcinoma involving adnexal tissue with low-grade morphology, as seen in this case. Endometrioid carcinoma of the fallopian tube can closely mimic FATPWO, particularly when PAX8 and CK7 are both negative. Molecular testing is limited in distinguishing between the two due to the absence of disease-defining alterations in FATPWO and the lack of specific mutations in some endometrioid carcinomas.

## Introduction

Female adnexal tumor of probable Wolffian origin (FATPWO) is a rare neoplasm believed to arise from mesonephric remnants in the adnexal region [1]. It occurs across a wide age range and is most commonly found in the paratubal soft tissue and broad ligament, with less frequent involvement of the ovary and retroperitoneum. Morphologically, FATPWO demonstrates a variety of architectural patterns and mild cytological atypia. To date, no specific immunohistochemical markers or genetic alterations have been definitively identified for this tumor.

The morphologic and immunophenotypic features of FATPWO can significantly overlap with other entities, including endometrioid adenocarcinoma and ovarian sex cord-stromal tumors. Retrospective studies have shown that FATPWO typically lacks expression of PAX8, PAX2, TTF1, and GATA3. It is variably positive for EMA, calretinin, inhibin, CK7, WT1, and CD10, and consistently positive for AE1/AE3, CAM5.2, and estrogen receptor (ER) [1]. A minority of cases have shown frequent KMT2D mutations, though the biological significance remains uncertain [2].

We report a challenging case involving a 51-year-old woman who underwent hysterectomy and right salpingo-oophorectomy for a large adnexal mass, ultimately diagnosed as endometrioid adenocarcinoma of the fallopian tube. The tumor’s morphology and immunoprofile mimicked FATPWO, including PAX8 and CK7 negativity, and lacked specific molecular alterations. Given the diagnostic difficulty, expert consultation was obtained. Based on the tumor's extensive tubular growth pattern and the possibility of PAX8 negativity in endometrioid carcinoma, the final diagnosis favored endometrioid carcinoma. The differentiation between FATPWO and endometrioid carcinoma is clinically critical due to their distinct behaviors and management strategies. FATPWO, a rare mesenchymal tumor of the adnexal region with low malignant potential, typically requires surgical excision with long-term surveillance due to infrequent recurrences or metastases. In contrast, endometrioid carcinoma is an aggressive malignant tumor of Müllerian origin, often necessitating extensive surgery and adjuvant therapies such as chemotherapy or radiation. Accurate histopathological and immunohistochemical diagnosis is essential to guide appropriate treatment and optimize patient outcomes.

## Case presentation

Clinical summary

A 51-year-old premenopausal woman presented with an adnexal mass. Her serum levels of human epididymis protein 4 (HE4) and cancer antigen 125 (CA-125) were within normal limits. She had no significant past medical history and no evidence of Peutz-Jeghers syndrome. Imaging revealed a large, complex, solid, and cystic lesion in the right adnexa, measuring up to 18 cm. A solid nodule measuring 5.3 × 4 cm was also seen along the left aspect of the cystic component, extending toward the left adnexa and likely associated with the primary right adnexal mass. Additionally, a complex cystic structure located posterior to the uterus, measuring 6.1 × 3.2 cm, was interpreted as a possible right hydrosalpinx.

Following gynecologic consultation, the patient underwent a simple hysterectomy, right salpingo-oophorectomy, omentectomy, peritoneal washings, and bilateral para-aortic and pelvic lymphadenectomy. Intraoperatively, a 20 cm freely mobile mass was noted, mostly solid with cystic components. The left ovary, left fallopian tube, and uterus were unremarkable. The omentum appeared normal, without nodularity or peritoneal implants. The intraoperative diagnosis for the adnexal mass was endometrioid adenocarcinoma.

Pathologic findings

Grossly, two masses measuring 20 × 19 × 10 cm and 11.5 × 4 × 3 cm were attached to a fragmented right fallopian tube. Both masses had mixed solid and cystic areas filled with hemorrhagic fluid. The cut surfaces were lobulated and tan-white with papillary architecture.

**Figure 1:**
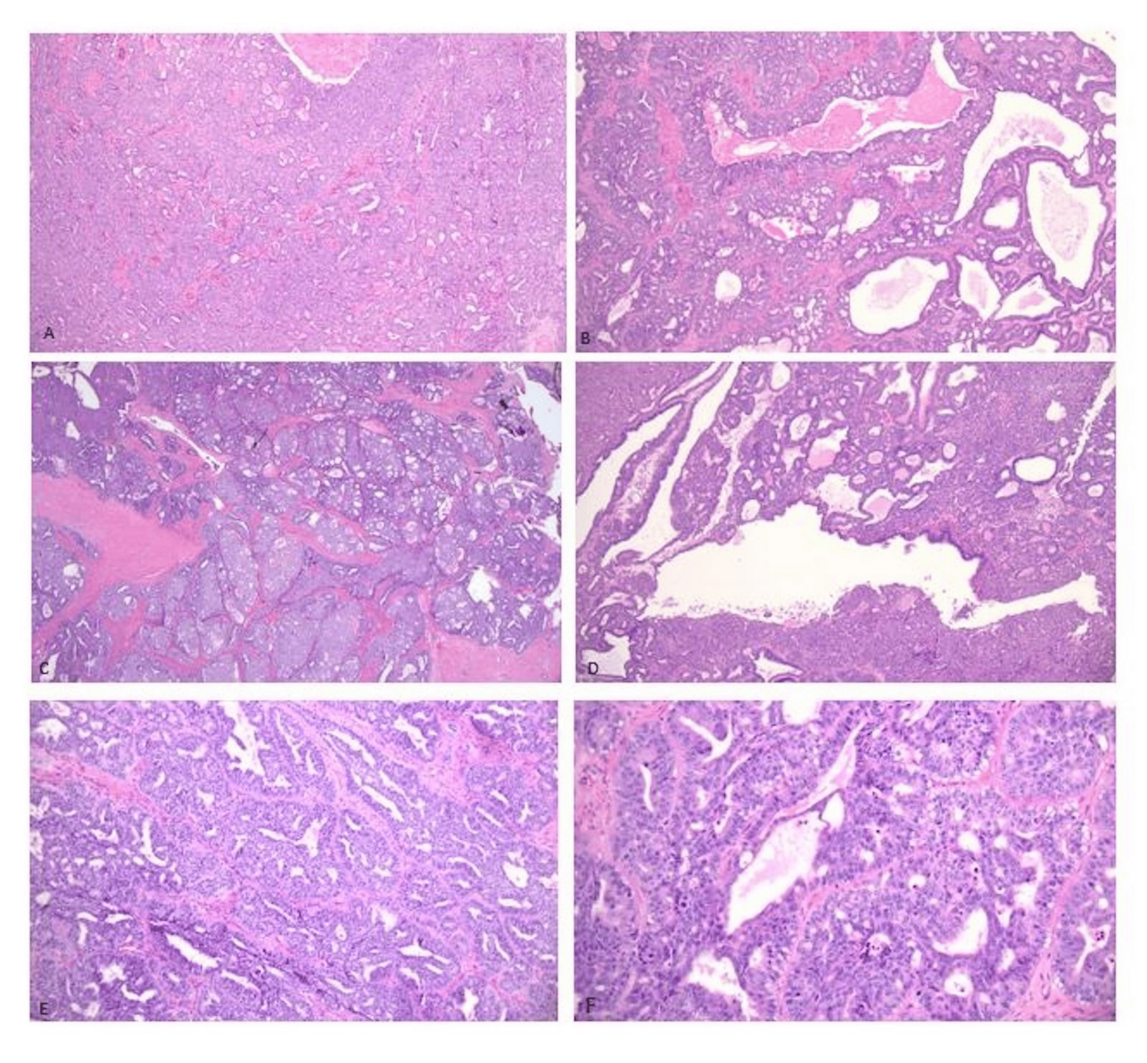
Hematoxylin and eosin stains. (A–D) Low magnification (40×) showing tumor cells forming compact tubular structures with eosinophilic secretions (A), cribriform architecture with sieve-like pattern (B), nested solid growth separated by delicate fibrous septa (C), and macrocysts with papillary projections (D). (E) Intermediate magnification (100×) and (F) high magnification (200×) views demonstrating stratified low cuboidal epithelial cells lining the tubules with prominent nucleoli.

Histologically, the tumor displayed diverse architectural patterns, including tubular, cribriform, papillary, and cystic growth with sieve-like luminal spaces containing eosinophilic secretions. The neoplastic cells were cuboidal with eosinophilic cytoplasm and variably prominent nucleoli. In some regions, the nuclei showed clearing and pale chromatin reminiscent of papillary thyroid carcinoma. Overall, the tumor exhibited low to moderate nuclear atypia and variable mitotic activity. Macrocystic areas with papillary projections, stromal hemorrhage, and edema were also noted. No squamous or mucinous differentiation was identified. A 1.5 mm microscopic focus of tumor involvement was seen in the right ovary. No necrosis, lymphovascular invasion, or perineural invasion was observed (Figure 1). The endometrium, ligaments, peritoneal washings, and omentum were negative for malignancy. All five pelvic and para-aortic lymph nodes were negative for metastatic carcinoma.

Immunohistochemically, the tumor cells were diffusely positive for CAM5.2, AE1/AE3, and ER; focally positive for CD10, EMA, vimentin, WT1, CDX2, and p16; and negative for PAX8, CK7, calretinin, GATA3, Napsin A, and TTF1. P53 showed wild-type expression. The Ki-67 proliferative index was approximately 20% in hot spot areas.

Molecular testing revealed no genetic alterations in MET, RET, PTEN, BRAF, EGFR, KRAS, or NRAS and no NTRK gene fusions. Microsatellite instability (MSI) testing was stable. Immunohistochemistry for PD-L1 and HER2 (gastric scoring) was negative. Importantly, no STK11 mutation was detected, excluding the possibility of STK11-related adnexal tumors. Taken together, the combination of extensive tubular architecture, focal papillary areas, low-grade morphology, and immunophenotypic findings supports a diagnosis of endometrioid carcinoma rather than FATPWO.

## Discussion

Endometrioid adenocarcinoma is a malignant epithelial tumor characterized by glandular differentiation and diverse architectural variants. While most commonly originating in the endometrium, it can also arise in other sites associated with endometriosis, such as the ovary, fallopian tube, and colon [3]. Its histologic variants include villoglandular, squamous, mucinous, clear cell, spindle cell, and sex cord-like patterns. These variants often pose diagnostic challenges, particularly when low-grade morphology overlaps with other rare tumors [3].

FATPWO is a rare neoplasm believed to derive from mesonephric remnants, most frequently in the paratubal region. Histologically, FATPWO exhibits combinations of solid, tubular, and sieve-like patterns, accompanied by eosinophilic luminal secretions and generally mild cytologic atypia. Immunohistochemically, FATPWO lacks specific markers, and its profile can overlap with multiple tumor types. In Bennett et al.'s study [4], the most common growth pattern was tubular (100%), followed by solid (73%) and sieve-like (47%), with eosinophilic secretions in 73% of cases. Tumor cells were positive for pancytokeratin and variably positive for EMA, GATA3, CD10, WT1, and ER. PAX8 positivity was rare (one of 15 cases). STK11 mutations were found in a subset with myxoid backgrounds, and other rare mutations (e.g., APC and MBD4) were identified in individual cases [2].

Hou et al.’s group [5] reported similar morphologic features in 11 FATPWO cases, all negative for PAX8 and GATA3, supporting a non-Müllerian, non-mesonephric origin. Goyal et al. [6] also found PAX8 negativity in eight FATPWO cases but consistent positivity in 18 ovarian/tubal endometrioid adenocarcinomas. Mirkovic et al. [2] found KMT2D mutations in 57% of FATPWO cases, but these were of uncertain significance, and no recurrent mutations (e.g., KRAS, NRAS, TP53, PTEN, and PIK3CA) or copy number alterations were identified.

In our case, the tumor displayed extensive tubular growth, low-grade cytology, and PAX8/CK7 negativity-features that strongly resemble FATPWO. However, molecular testing revealed no definitive genetic alterations, and the tumor lacked hallmark mutations seen in other mimics. Although some nuclei showed PTC-like chromatin clearing, negative staining for TTF1 and GATA3 and the absence of KRAS/NRAS mutations ruled out mesonephric-like adenocarcinoma [7]. The tumor also lacked STK11 mutations, arguing against STK11-related adnexal tumors, which are typically high-grade, aggressive, and associated with Peutz-Jeghers syndrome [8].

While PAX8 loss is more common in high-grade endometrial carcinomas [9], this case was well-differentiated. Travaglino et al. [10] described ovarian endometrioid carcinomas with sex cord-like differentiation showing PAX8 loss and expression of CK7, EMA, and CDX2. Our case, however, lacked sex cord-like features and a matching immunoprofile.

Interestingly, no prior literature has reported papillary architecture in FATPWO. In contrast, our case showed focal papillary growth with macrocyst formation and stromal hemorrhage, possibly reflecting prior endometriosis. However, no definitive histologic evidence of endometriosis was present, likely due to the tumor's large size replacing the precursor lesion entirely.

In summary, this case represents a rare instance of low-grade endometrioid carcinoma arising in the fallopian tube that closely mimics FATPWO both morphologically and immunophenotypically. The absence of PAX8 and CK7 expression and the lack of disease-defining molecular alterations highlight the diagnostic challenge. This case underscores the importance of recognizing that endometrioid carcinoma can masquerade as FATPWO and that loss of PAX8 and CK7 does not preclude a diagnosis of endometrioid carcinoma in the adnexal region.

## Conclusions

This case illustrates the significant diagnostic overlap between low-grade endometrioid carcinoma and FATPWO, particularly when immunoprofiles and morphologic features converge. Despite the absence of hallmark mutations and typical immunomarkers such as PAX8 and CK7, the overall architecture, clinical setting, and subtle histologic cues support a diagnosis of endometrioid carcinoma of the fallopian tube. Recognition of this mimicry is crucial, as reliance on limited markers or the absence of molecular alterations may lead to misclassification. This case expands the known morphologic spectrum of endometrioid carcinoma and highlights the importance of integrating histology, immunohistochemistry, and clinical context when diagnosing adnexal tumors.
